# Social Values and Determinants of Cultural Fit in Quebec: The Roles of Ancestry, Linguistic Group, and Mental Health Status

**DOI:** 10.3389/fpsyg.2019.00287

**Published:** 2019-05-07

**Authors:** Daina Crafa, Joanna Q. Liu, Mathieu B. Brodeur

**Affiliations:** ^1^ Integrated Program in Neuroscience, McGill University, Montréal, QC, Canada; ^2^ Interacting Minds Centre, Aarhus University, Aarhus, Denmark; ^3^ Department of Psychology, McGill University, Montréal, QC, Canada; ^4^ Department of Psychiatry, Douglas Mental Health University Institute, McGill University, Montréal, QC, Canada

**Keywords:** cultural fit, cultural membership, cultural consensus, Canadian linguistic groups, social values schema, self-construal, intersectionality

## Abstract

Many quantitative cross-cultural research studies assume that cultural groups consist of anyone born and raised in the same country. Applying these criteria to the formation of study samples may produce cohorts that share a country but are heterogeneous in relevant domains of culture. For example, in Canada, Franco- and Anglo-Canadians are generally assumed to represent different linguistic groups but the same cultural group. However, speaking a different first language also can mean exposure to different media, information, and conventions, which are known to shape certain cultural domains, such as social values. Other factors may also produce cultural heterogeneity. For example, ancestral origins and recency of familial migration may influence endorsed social values after exposure to diverse cultures or norms. Mental health status or psychiatric conditions may also influence subscription of social values due to different lifestyle demands. Understanding the nuanced contributions of diverse backgrounds to cultural membership and fit (i.e., the degree to which an individual behaves like other cultural members) is useful when performing quantitative cross-cultural studies to minimize alternative explanations for statistical outcomes. This study used Cultural Consensus Analysis (CCA) to assess the cultural fit of social values for 222 Canadians, who had participated in cross-cultural neuropsychological experiments. CCA is an anthropological statistical method for evaluating cultural agreement of a sample. Participants were systematically evaluated by linguistic groups (French and English), migratory generation (1st–3rd+), and mental health status (healthy and patient). Group and individual variances were statistically interrogated. Results demonstrated that Franco- and Anglo-Canadians represent different cultural groups cohabitating in Quebec. Social values dividing Franco- and Anglo-Canadians were also identified. Second and third generation Canadians held more heterogeneous social values than Canadians, whose families had migrated earlier. Second generation Canadians with psychiatric disorders showed notably reduced cultural fit with other Canadians, which supports other literature reporting difficulties experienced by second generation migrants. However, third and later generations of Canadians with psychiatric disorders held a greater range of social values compared to healthy Canadians but still were good fits for Canadian culture. This study concluded that linguistic group and migratory generation partially determines cultural group for the social values domain while mental health status does not, contrary to theories proposed by previous literature.

## Introduction

*Culture* is a multidimensional concept, and cultural group membership depends on how membership is defined and which domain of culture is being considered. *Cultural membership* describes whether an individual meets basic traits ascribed to a cultural group while *cultural fit* is a more specific term referring to the degree to which an individual behaves like others within that group within the specific cultural domain (Mokros, 2003; Sam and Berry, 2010). Because within-group cultural differences can be difficult to measure statistically, quantitative studies sometimes define membership according to superficial traits (e.g., nationality or linguistic group) and assume that their study samples are “a good fit” for a culture by virtue of this superficial membership. For example, many studies assume that individuals are cultural members because they are born and raised within specific geopolitical borders (Cohen, 2009; Cole, 2009). However, this manner of defining cultural membership conflates country with culture, and might not sufficiently account for potential diversity (e.g., intersectional memberships, variations in fit) that a country’s native residents may embody (Cohen, 2009). Even when more specific definitions of cultural membership are applied, an individual may reflect their cultural group to varying degrees and in varying domains, and multiple domains are conflated in many studies (Mobasseri et al., 2017). Therefore, characterizing cultural fit within a single cultural domain, such as social values, may be useful for understanding one dimension of the relationship between country and culture. To examine social determinants of within-culture cohesion, this study applies Cultural Consensus Analysis (CCA), which is an anthropological method of defining cultural group membership, to the domain of social values using carefully defined study samples.

Culture can be defined across multiple domains, and social values represent one deeply-rooted domain of culture to which members may or may not subscribe (Hofstede, 2001; Schwartz, 2006). Social values vary substantially across cultural groups and are considered as a defining feature of group memberships (Elfenbein and O’Reilly, 2007). They are passed down vertically and obliquely between generations and horizontally among peers, and they provide foundations for complex, symbolic cultural behaviors, such as manners and traditions (Trommsdorff, 2009; Bisin and Verdier, 2010). Traditionally, the domain of social values has been studied by examining self-construal groups. *Self-construal* is a classification system defining social value orientations that define people as either independent, meaning that they are oriented toward individuality and value personal preferences, or interdependent, meaning that they are oriented toward community and value group benefits (Markus and Kitayama, 1991; Triandis, 1995; Oyserman et al., 2002). Self-construal has been widely criticized for being overly simplistic and binary (Mateo et al., 2013). *Social values schema* (SVS) describes the constellation of social values that an individual holds to varying degrees as represented on a continuum (Osland et al., 2000; Cheng and Fleischmann, 2010; Boer and Fischer, 2013; Bilsky et al., 2015). SVS is correlated with *self-construal*, butrepresents a distinct cultural domain (Crafa et al., 2018). While self-construal measures self-reported actions and social orientations, SVS measures the degrees of valuations that partiallyconstruct self-identity (e.g., “I do” vs. “I am” statements). The SVS is additionally more nuanced than measures of self-construal because it considers multiple social values and the degree to which an individual endorses or rejects each value, thus resulting in a constellation of social value preferences rather than resorting to binary classifications (Crafa et al., 2018). For example, an individual may value agreeableness and independence or assertiveness and interdependence – or all four social values to varying degrees. Examining SVS allows the scope of these nuances and ostensible contradictions to be captured without invoking binary categories (Crafa et al., 2018). Thus, we propose that SVS can be used to measurehomogeneity ofcultural membership and fit.

Many quantitative research studies classify sociocultural groups within countries according to language or familial ancestry instead of culture (e.g., Yang et al., 2011; Ellefson et al., 2017; Kito et al., 2017; Priego-Valverde et al., 2018; for review see Park and Huang, 2010); however, recent studies demonstrate that significant cultural gaps emerge within countries as well as between them (McSweeney, 2009; Taras et al., 2016). Most countries house multiple linguistic and ancestral groups whose members may have adopted the country’s dominant culture to varying degrees. Even if their ancestors have been in the country for multiple generations, members of these ancestry groups may feel proud of their heritage and maintain some of its cultural values and traditions (Gans, 2014). For example, Italian-Americans and Irish-Americans in the United States represent the descendants of distinct groups of migrants, most of whom immigrated circa 1900; descendants have assimilated to United States culture but still maintain some of the languages, traditions, and social values from their ancestral countries (Luconi, 2007). Likewise, the French-speaking population in Louisiana in the United Statesretains their own, unique culture (Le Menestrel, 2014). Many descendants of the indigenous Ainu people, who originated in regions that are now geopolitically-defined as parts of Japan and Russia, maintain their distinct culture while other group members have fully assimilated into their new culture due to past integration efforts initiated by the respective governments (Shibatani, 1990; Fogarty, 2008). Groups such as these may be treated as members of their country’s culturein if demographic study forms do not ask whether someone’s great grandparents immigrated through Ellis Island, for example – they may be considered a cultural member although an individual’s cultural fit may lean more toward their ancestral culture and not the dominate culture of their birth country.

The population in Canada provides an interesting opportunity to interrogate the relationship between country, language, ancestry, and cultural fit. Canada has two national languages representing people from diverse backgrounds and migration histories. Canada houses numerous Franco-Canadians who are descendants of French colonists (i.e., Quebecois), whose immigration from France may date back to the 17th century, or of migrants from French territories, such as Haiti or Martinique, which have distinct cultural lineages of their own (Landry et al., 2010). Canada also houses numerous Anglo-Canadians who are defined primarily by their linguistic heritage and have ancestors from a diverse array of countries (Day, 2000; Wood and Gilbert, 2005). Franco- and Anglo-Canadians are demarcated partially by native language and partially by ancestry and therefore potentially contain diverse cultural memberships within each group. In part because of this ancestral diversity, Franco- and Anglo-Canadians are often assumed to represent two different linguisticgroups within Canadian culture, although many differences in traditions and heritage identities exist between groups (Arias-Valenzuela et al., 2016). Some studies acknowledge cultural differences in Franco- and Anglo-Canadians without directly evaluating whether these groups can more accurately be described in terms of homogeneous cultures or linguistic differences (see e.g., Sussmann and Rashcovsky, 1997; Williamson and Harrison, 2010).

Defining Franco- and Anglo-Canadians as linguistic groups under a single umbrella culture assumes that language does not substantially influence cultural values, while defining them as separate cultural group risks ignoring the magnitude of the dominant culture for providing a common cultural framework. Cultural differences can of course emerge among groups that share a language buthave other differences; however, languageacts as a filter for specific types of cultural information. For example, one’s native language may influence the prevalence and accessibility of information. Although these linguistic groups reside in the same country, they may be exposed to very different social values, customs, and expectations through friends and media depending on their native tongue and prescribed group membership (Pavlenko and Lantolf, 2000; Chiesa et al., 2012). Because of these divergent experiences, linguistic group members may also have divergent SVS. These linguistic groups are sometimes combined or conflated with fluency or verbal competencein empirical literature that may be influenced by social values, such asstudies of identity formation and socialization behaviors (see e.g., Richard et al., 2008; Hope et al., 2014; Lemelin et al., 2014; Ethridge et al., 2017). Linguistic group, of course, does not necessarily equal cultural homogeneity. However, Franco- and Anglo-Canadians also have different ancestral lineages, many of which have immigrated into the country within recent generations and both have been granted equality as national languages. These circumstances raise the question of whether Canada has a unified, overarching culture regardless of language or lineage, or whether Canada houses two groups that are distinct in certain cultural domains due to the strength of these influences.

Therefore, the *cultural consensus* of Canadians, i.e., their relative agreement with each other, is useful to assess. CCA is a statistical approach used primarily by anthropologists that relies on factor analysis to construct a cultural model from a population’s consensus and evaluate the relative cultural fit of group members therein (Weller, 2007). CCA can identify cultural differences between groups as well as subcultures within groups (Caulkins, 2004). The focus of CCA is generally on model construction, which takes a bottom-up approach by including members of predefined cultural groups. However, a top-down approach also can be used to assess whether a group of people have sufficient consensus to constitute a cultural group (Caulkins and Hyatt, 1999). In this respect, CCA is arguably a useful tool for quantitative studies, e.g., in psychology or neuroscience, to assess whether study participants reflect a unified cultural group in the cultural domain of inquiry without having to perform between-group comparisons. For example, the diverse ancestral backgrounds of Anglo-Canadians may play significant roles in determining cultural group membership. Top-down CCA uses predefined statistical thresholds to determine whether the contributions of ancestral diversity are minimal compared to the contributions of their linguistic group, which can be applied to identify similarities and differences between these linguistic groups (Weller, 2007).

Accordingly, Franco- and Anglo-Canadians may represent two distinct groups in the domain of social values and CCA can be applied to a random sample of participants to assess their relative consensus (Hruschka and Maupin, 2013; McDonald, 2014). However, since Anglo-Canadians have widely diverse ancestral backgrounds, most of which have immigrated recently, it is also possible that Franco-Canadians will represent a culturally defined group while Anglo-Canadians will show a greater range of cultural backgrounds. Therefore, the contribution of familial migratory generations must also be evaluated for this cohort. In first generation migrants, cultural fit is associated with the relative degrees of affiliation with their home and receiving cultures (Berry, 2005; Schiefer et al., 2012). However, the cultural fit of later generations of descendants have been less frequently studied (see e.g., Jasni et al., 2018). Residual traits, such as age or gender, may also systematically influence cultural fit and CCA can be used to evaluatehomogeneity amongthese influences as well (Caulkins, 2004).

While linguistic skills may grant or preclude access to cultural information, social competence may likewise influence the cultural information a person is exposed to. Psychiatric disorders represent an extreme on the spectrum of social competence. Approximately, 20% of Canadians will suffer from a psychiatric diagnosis during their lifetime (Smetanin et al., 2011). Most psychiatric diagnoses are defined by inappropriate social behaviors or by difficulty processing social information, and these challenges may influence cultural fit or membership (Chen and French, 2008). For example, schizophrenia spectrum disorders are typically lifetime psychiatric conditions associated with both inappropriate social behaviors and difficulty processing social information (Mäkinen et al., 2008; Grant and Beck, 2010). Individuals diagnosed with schizophrenia are assumed to have poor cultural fit, but the cultural fit of patients diagnosed with schizophrenia has not been statistically interrogated, althoughseveral studies have demonstrated that lifestyle, social support, stigma, and mental health status can contribute to cultural fit (Dressler et al., 2007). Many patients with schizophrenia report experiencing similar hardships due to stigma and social difficulties, which could conceivably influence their SVS (Piff et al., 2010). Patients may share more social values in common with their larger cultural group or they may share more in common with other patients (Smith et al., 2004; Rashed, 2013). In most populations, second generation migrants experience high rate of mental health problems and significant feelings of alienation (see e.g., Pumariega et al., 2005; Bourqueet al., 2011), although one study shows that this might be less pervasively true for Canadians (Dealberto, 2010). Identifying whether poor cultural fit is a possible cause of stress and alienation may yield valuable insight into the reasons for these higher rates. CCA has been previously proposed as a relevant clinical tool and provides an opportunity to examine the relationship between culture, language, and mental health (Smith et al., 2004).

The present study assesses the contributions of native language, migratory generation, and psychiatric status to cultural membership in the domain of social values. First, the social values of healthy Franco- and Anglo-Canadians were assessed to characterize domain-specific cultural membership and fit. Since Anglo-Canadians have substantially diverse ancestral backgrounds, migratory generations in this cohort were also evaluated. Patients with schizophrenia were subsequently compared across language groups to assess whether patients share a common culture with other patients or whether they have good cultural fit with “healthy” language group members. Finally, the co-contribution of ancestry was assessed in this cohort to determine whether migratory generation influenced cultural membership or fit. We found that Franco- and Anglo-Canadians represent inhomogeneous cultural groups.

## Materials and Methods

### Participants

All study participants (*N* = 222) were between the ages of 18–50. To minimize environmental confounds and isolate differences related to language, familial ancestry, and mental health status, all participants were born and raised in Canada by their biological parents and living in Montreal at the time of the study. Complete demographic information appears in Table 1. This study was carried out in accordance with the recommendations of the Research Ethics Boards of the Montreal Neurological Institute and of the Douglas Mental Health Institute with written informed consent from all subjects. The protocol was approved by the Research Ethics Boards of the Montreal Neurological Institute and of the Douglas Mental Health Institute. All subjects gave written informed consent in accordance with the Declaration of Helsinki.

**Table 1:** Participant demographics.

*Participant Demographics*									
Cohort	Total N *(%female)*	Mean age *(SD)*	Self-construal N INDE /N INTER	Foreign grandparents % *(N)*	FR/EN Bilingual % *(N)*	Mean education Years *(SD)*	Mean SES CAD *(SD)*	FSIQ *(SD)*	SAPS *(SD)* / SANS *(SD)*
Healthy Franco-Canadians with Canadian Parents	70 *(47.1%)*	29.0 *(8.4)*	53 / 17	4.3% *(3)*	92.9% *(65)*	12.9 *(1.9)*	35,807 *(28,784)*		
Healthy Anglo-Canadians with Canadian Parents	77 *(46.8%)*	28.5 *(9.3)*	49 / 28	61.0% *(47)*	79.2% *(61)*	14.3 *(2.1)*	41,769 *(38,737)*		
Healthy Canadians with Foreign Parents	30 *(60.0%)*	26.6 *(9.5)*	19 / 11	100.0% *(30)*	66.7% *(20)*	11.9 *(2.6)*	41,561 *(52,565)*		
Matched Franco-Controls with Canadian Parents	17 *(41.2%)*	37.2 *(6.8)*	15 / 2	5.9% *(1)*	94.1% *(16)*	12.0 *(3.4)*	43,235 *(31,172)*	107 *(16)*	
Matched Franco-Patients with Canadian Parents	17 *(23.5%)*	36.9 *(6.8)*	10 / 7	0.0% *(0)*	94.1% (16)	12.9 *(3.0)*	10,118 *(6,669)*	99 *(12)*	9.6 *(5.8)* / 13.1 *(5.3)*
Matched Anglo-Controls with Canadian Parents	16 *(12.5%)*	36.0 *(7.0)*	14 / 2	62.5% *(10)*	93.8% *(15)*	14.9 *(1.7)*	37,333 *(22,902)*	109 *(12)*	
Matched Anglo-Patients with Canadian Parents	16 *(6.3%)*	38.9 *(7.4)*	10 / 6	56.3% *(9)*	81.3% *(13)*	13.3 *(2.7)*	17,813 *(10,641)*	98 *(18)*	7.5 *(4.1)* / 11.2 *(5.2)*
Matched Patients with Foreign Parents	12 *(33.3%)*	31.5 *(9.0)*	3 / 8	100.0% *(30)*	100.0% *(12)*	13.5 *(1.0)*	32,500 *(29,640)*	110 *(12)*	
Matched Patients with Foreign Parents	12 *(33.3%)*	36.3 *(7.3)*	3 / 8	100.0% *(30)*	100.0% *(12)*	12.5 *(3.0)*	15,000 *(7,071)*	92 *(8)*	9.0 *(4.0)* / 8.9 *(7.5)*

This table depicts the cohorts of Canadians that were included in the study. A total of 267 Canadiana participated in this study and were grouped according to dominant language, parental migration status, and psychiatric diagnosis. Healthy Canadians were divided by linguistic (Franco and Anglo) and ancestral groups (Canadian and Foreign parents). The matched cohorts indicate groups of healthy control participants matched with patients from the same linguistic and ancestral backgrounds.

Abbreviations: N = total count, SD = standard deviation, INDE = independent self-construal, INTER = interdependent self-construal, FR = French language, EN = English language, CAD = Canadian dollars, FSIQ = full scale intelligence quotient, SAPS = Scale for Assessment of Positive Symptoms, SANS = Scale for Assessment of Negative Symptoms. The SAPS and SANS are standard clinical scales quantifying patients’ symptom severity (see Andreasen, 1984a, 1984b for full discussion).

This study broadly consisted of four analysis groups: Healthy Canadians with Canadian Parents, Healthy Canadians with Foreign Parents, Matched Patients-Controls with Canadians Parents, and Matched Patients-Controls with Foreign Parents. Participants were subdivided into linguistic groups according to their native language. Fluency in other languages was also recorded.

The cohorts of *Healthy Canadians with Canadian Parents* included 70 Franco- and 77 Anglo-Canadians with no history of psychiatric disorders. All Healthy Canadians were raised by parents who were also born and raised in Canada. The countries where family members were born and raised were recorded, but could not be controlled past the parents’ generation. Canada is in the top 25 destinations for migrants (Migration Policy Institute, 2015) and has relatively high rates of migrations compared to global trends: 21.9% of total population were born outside of Canada according to 2016 census; 8.1% of adults are second generation Canadians; and 57.6% are third generation or greater. They are largely English-speakers (23.8% Anglophone and 3.4% Francophone). In Canada, a disproportionate percentage of Anglo-Canadians (up to 62.5%) have grandparents who were born or raised outside Canada compared to Franco-Canadians (up to 5.9%), and 17.9% of the total Canadian population is bilingual (Statistics Canada, 2016).

A cohort of second generation migrants, i.e., *Healthy Canadians with Foreign Parents* (*N* = 30), with no history of psychiatric disorders was also included in this study. No Francophone respondents had foreign-born parents, so all participants in this cohort spoke English as the native and primary language, and were born and raised in Canada to parents who were born and raised in another country. The purpose of including this group was twofold. On one hand, since CCA is still a relatively new statistical approach, including the group of Healthy Canadians with Foreign Parents ensured that CCA was sensitive enough to distinguish between cohorts of individuals who had been born and raised in Canada. Numerous studies have reported that many bicultural individuals simultaneously hold the social values of the dominant culture in their birth country and of their parents’ culture, and it follows that this cohort would be more heterogeneous compared to Canadians who were raised by Canadian parents (Berry, 1980; Lee et al., 2006; Yoon et al., 2011). On the other hand, inclusion of this cohort also ensured that results in the main analyses were due at least in part to linguistic differences, and not to ancestral heterogeneity alone. This cohort was compared to the Healthy Canadians with Canadian parents, who were divided by migratory generation according to whether they had foreign grandparents (i.e., third generation migrants) or whether their familial migration occurred earlier (i.e., fourth generation migrants or greater).

*Matched Patients-Controls with Canadians Parents* consisted of 17 Franco- and 16 Anglo-Canadians, all of whom were raised by Canadian parents and diagnosed with chronic schizophrenia (>5 years) according to DSM-5 criteria. The patient cohort was matched by age and sex to 17 Franco- and 16 Anglo- Canadians from the Healthy Canadians cohort. In addition to having no history of psychiatric illness, the matched controls had no evidence of potentially undiagnosed psychiatric disorders according to the Structured Clinical Interview for DSM-5 (SCID; First et al., 2015). The patients and controls were also matched as closely as possible by intelligence quotient (IQ) measured by the Wechsler abbreviated scale of intelligence second edition (WASI-II; Wechsler and Hsiao-pin, 2011), years of education and household socioeconomic status. Compared to healthy participants, individuals with schizophrenia have difficulty in completing IQ testing due to side effects of their symptoms or medications and they disproportionately receive social economic assistance; group differences in this study are comparable to other studies (Walterset al., 2010). Medications were also recorded but too disparate to be statistically evaluated. In the patient cohort, active symptoms at the time of testing were measured using Scale for the Assessment of Positive/Negative Symptoms (SAPS/SANS; Andreasen 1984a,b), which is a standard clinical assessment that rates the symptom severity on a scale of 0–5 according to 9 domains: hallucinations, delusions, bizarre behaviors, thought disorders (SAPS); affective blunting, alogia, avolition, asociality, and attention (SANS).

The cohort of *Matched Patients-Controls with Foreign Parents* included 12 Anglo-Canadian patients matched by parents’ birth countries to 12 Healthy Anglo-Canadians with no histories of psychiatric disorders and no evidence of potentially undiagnosed disorders *via* the SCID. They were also matched as closely as possible by age and gender. IQ, years of education, household socioeconomic status, and symptom severity ratings and medications were also recorded.

**Figure 1:**
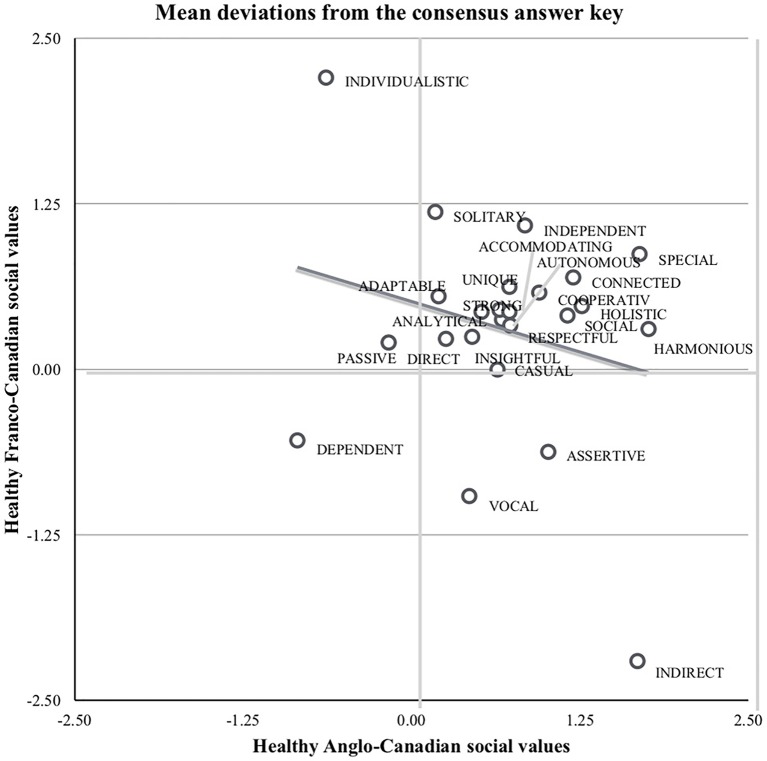
Adjusted ratings depict patterns of social values across Canadian cultural groups. When considering culture, the varying degrees to which social values are held characterize the priorities of group members and degrees of differences between groups. The constellations of social values across Canadian cultures according to the CCA factor dimension illustrate distinct patterns between Franco- and Anglo-Canadians.

**Table 2:** T-test comparisons further describe patterns of social values across Canadian cultural groups. Independent samples t-tests quantify observed groupwise differences. This table on the right depicts the varying degrees to which Franco- and Anglo-Canadian subscribe to each social value relative to each other, adjusted for their shared cultural background.

*T-Test Results for Healthy Canadians*			
Social Value	T-test Results	Anglo-Canadian Mean *(±SD)*	Franco-Canadian Mean *(±SD)*
Assertive	t(144) = 3.99, p < 0.001**	1.01 *(2.91)*	-0.63 *(1.88)*
Harmonious	t(144) = 3.65, p < 0.001**	1.76 *(2.87)*	0.31 *(1.76)*
Individualistic	t(144) = -5.89, p < 0.001**	-0.64 *(2.36)*	2.20 *(3.41)*
Vocal	t(144) = 2.94, p < 0.001**	0.43 *(3.11)*	-0.96 *(2.51)*
Holistic	t(144) = 1.52, p = 0.001**	1.26 *(3.29)*	0.48 *(2.69)*
Indirect	t(144) = 8.31, p = 0.001**	1.68 *(2.42)*	-2.20 *(3.20)*
Special	t(144) = 1.70, p = 0.026*	1.70 *(3.12)*	0.87 *(2.69)*
Connected	t(144) = 1.09, p = 0.031*	1.97 *(3.09)*	0.69 *(2.42)*
Adaptable	t(144) = -1.32, p = 0.052	0.21 *(1.44)*	0.55 *(1.97)*
Solitary	t(144) = -1.97, p = 0.068	0.18 *(2.98)*	1.19 *(3.21)*
Independent	t(144) = -0.76, p = 0.098	0.84 *(1.72)*	1.09 *(2.18)*
Respectful	t(144) = 1.66, p = 0.120	0.73 *(1.69)*	0.33 *(1.14)*
Social	t(144) = 1.73, p = 0.124	1.16 *(2.83)*	0.40 *(2.37)*
Strong	t(144) = 0.55, p = 0.271	0.65 *(2.40)*	0.45 *(1.96)*
Cooperative	t(144) = 0.97, p = 0.290	0.95 *(2.51)*	0.58 *(2.02)*
Casual	t(144) = 1.35, p = 0.297	0.64 *(2.72)*	-0.00 *(2.95)*
Accommodating	t(144) = 0.87, p = 0.482	0.73 *(2.24)*	0.44 *(1.78)*
Unique	t(144) = 0.29, p = 0.500	0.73 *(2.08)*	0.62 *(2.17)*
Analytical	t(144) = 0.26, p = 0.522	0.52 *(1.90)*	0.43 *(1.97)*
Insightful	t(144) = 0.79, p = 0.586	0.45 *(1.67)*	0.25 *(1.42)*
Autonomous	t(144) = 1.06, p = 0.798	0.67 *(1.75)*	0.38 *(1.61)*
Passive	t(144) = -0.67, p = 0.878	-0.17 *(3.31)*	0.20 *(3.43)*
Direct	t(144) = 0.05, p = 0.879	0.26 *(2.54)*	0.23 *(2.58)*
Dependent	t(144) = -0.55, p = 0.974	-0.85 *(3.35)*	-0.33 *(3.45)*

### Social Values Schema Scale

The Sasta Social Values Schema Scale (SVS scale; Crafa et al., 2018; available at www.dainacrafa.com/sasta) is a validated self-judgment questionnaire modeled after experimental tasks used by quantitative cross-cultural studies (e.g., Chiao et al., 2010; Ng et al., 2010; Ray et al., 2009). The SVS scale consists of 12 general self-descriptions (“In general, I am”) followed by a word associated with independent social values and 12 contextual self-descriptions (“When I am with my mother, I am”) followed by an interdependent social values word. The complete word list is depicted in Figure 1, Table 2. The different formulations of the questions are designed to mimic relational differences of the independent and interdependent selves that have been used in previous studies (e.g., Chiao et al., 2010); however, it represents a distinct cultural domain from self-construal by measuring values that constitute self-identity rather than behavior-based social orientations (Crafa et al., 2018). Participants rate on a 10-point Likert scale how well a social value described them (from −5 extremely poorly to 5 extremely well, with no neutral “0” option). Unlike other self-judgment measures, the SVS scale does not categorize participants into groups. Instead, it assumes that most people hold a combination of independent and interdependent values, and measures proportional subscriptions to these values individually. The SVS scale has previously been replicated and validated across multiple cohorts and applied to patient groups (Crafa et al., 2018). The present study examined the individual Likert ratings for each social values’ words on the SVS scale. Although CCA questionnaires often rely on “we-intentionality” phrasing, the SVS scale uses “I-intentionality” phrasing, in order to examine individual variations in value schema rather than social perceptions of cultural values schemas. We believe this is more useful for applying outcomes of the present study to quantitative cross-cultural studies, which may need to characterize cultural agreement for a variety of questionnaires; other clinical studies have applied “I-intentionality” formulations to CCA and other cultural examinations (Smith et al., 2004; Chiao et al., 2010; Elsenbroich, 2014; Zahavi, 2015).

### Self-Construal Scale

The Self-Construal Scale (Singelis, 1994) contains 30 questions (e.g., “I am unique”) rated on a seven-point Likert scale (strongly agree to strongly disagree). Scores are calculated by subtracting interdependence from independence values (Kitayama et al., 2014). Participants with a positive score were classified as having primarily independent self-construal and participants with a negative score were classified as having primarily interdependent self-construal (cf. Chiao et al., 2010).

### Translation Procedures

Translation is an important process in any cross-lingual study to ensure that all versions of the study scales have similar linguistic and cultural meanings (Beaton et al., 2000; Su and Parham, 2002). The original versions of all scales were in English. Each scale was first translated into Canadian French by two bilingual research team members who had been born and raised in Montreal. A third bilingual speaker then back-translated each scale into English. Two bilingual research team members who had not been involved in the original translations reviewed the translations. A pilot cohort of 10 French-speaking Canadians completed each scale, and at the end, they were asked to comment on the clarity, commonality, and content of the language.

Since the SVS scale contains focal words that would be used for CCA, frequency of use between the focal words across languages was also assessed. To minimize differences in meaning and confirm consistency of prevalence of all 24 English/French word pairs in the corresponding language, language was standardized between English and French corpus frequencies. This was done by comparing the Français Contemporain (PFC) corpus^1^, whichrepresents the word frequencies of Quebec French, and the Corpus of Canadian English^2^. The frequency unit was standardized between the two corpuses by adjusting to word per million. The standard deviation between word frequency per million of the English and French word pairs was calculated, and pairs differing by 2 or more standard deviations were revised to find a pairing with greater commonalities.

### Evaluation of Cultural Consensus and Fit

Essentially, CCA quantifies (1) whether group agreement is sufficient to constitute a cultural model of a specific domain and (2) how well each individual fits within that cultural model. Because CCA is descriptive, it can be applied to a group many times without risk of positive findings due to probability biases, thus allowing thorough interrogation of group differences and characteristics by combining different groupings of participants (Hruschka and Maupin, 2013; McDonald, 2014).

The informal data model of CCA was applied to SVS data using SPSS version 24 for Mac (IBM Corp, 2016). The informal data model of CCA is a statistical approach that applies data reduction, principal components analysis (PCA) in the present study, to Likert scale responses to generate a statistical model of a cultural domain and assess cultural fit for each group member individually (Weller, 2007). Previous studies have reported no statistical differences between using PCA versus the alternative approach, minimal residual factor analysis (Hume, 2012). The first two factors are extracted *via* PCA. If cultural consensus is achieved (i.e., if a cohesive model has successfully been generated), the eigenratio between the first and second factors of the PCA should be ≥3:1 (Romney et al., 1986). Individual factor loadings are generated for each participant that indicate cultural fit, with higher values corresponding with greater agreement with other group members [i.e., cultural competence coefficient, Romney et al. (1986)].

This study took a top-down approach to assessing cultural consensus, in which all recruited participants were included in the analysis, to evaluate whether sufficient agreement across participants emerged (i.e., does the randomly constructed group achieve a factor ratio of ≥3:1). Participant groups were defined as having high cultural fit overall if they achieved group validity (≥0.95) and had a high cultural consensus (≥3:1). Low cultural consensus (≤3:1) indicates poor fit of participants based on grouping parameters.

The minimum sample sizes for CCA are generally determined by the consistency of consensus across the group. The Spearman-Brown Prophecy Formula is applied post hoc, using the mean Pearson correlation coefficient to determine sample validity; target validity level is 0.95 or higher (Romney et al., 1988; Weller, 2007). Samples can only achieve validity if they also have consensus (Weller, 2007). This approach is not practical when using a top-down approach to evaluate whether a population demonstrates consensus ina specific cultural domain. Groups that do not have consensus will not achieve validity due to the circular construction of the formula.

To circumvent this problem, in the present study cohorts that might not achieve consensus (e.g., patients) were matched with cohorts that should (e.g., controls) to determine whether the sample size is large enough for consensus to be achieved. In this case-control design, the matched controls acted as statistical standards demonstrating whether cultural consensus and group validity can be achieved at that N-value. This approach has the added benefit of ensuring group consistency, whereas previous studies of patient cohorts that perform CCA without matching potentially leave significant social variables unaccounted for (Smith et al., 2004). First, participants were matched according to demographic traits. Second, the control cohorts were assessed for sufficient consensus (≥3:1). Third, the case cohorts were evaluated individually at first and then combined with the control cohort to assess, first whether the case cohort is disparate or achieves consensus on its own and then to assess whether the case cohort participates in the broader Canadian culture.

Individual variations in cultural fit were then assessed by correlating component score loadings corresponding with the two factors generated by the PCA with continuous demographic traits (age, SCS score) using Pearsons’s test and comparing nominal groups (gender) using independent sample t-tests. Significant factor 1 results account for cultural groupings in social values, indicating that participants display systematic groupwise differences in their value schemas; while significant factor 2 results indicate possible subcultural groupings by reflecting secondary patterns in the underlying data structure (Boster and Johnson, 1989; Dressler et al., 2014).

### Assessment of Divergent Social Values Between Groups

Relative differences between the Franco- and Anglo-Canadian groups were first adjusted for shared background by creating an “answer key” for the tested model by averaging the scores of participants who had an average competency (i.e., shared responses with other participants using the PCA Component Matrix output table generated by SPSS) above 0.5 and all positive competency scores (for detailed methods see Dressler et al., 2014). Adjusted Likert ratings for each social value word on the SVS were compared across linguistic group using independent sample t-tests to determine which social values differed between healthy Franco- and Anglo-Canadians with Canadian Parents. This test was not performed on the familial migration cohorts since variations in these results may be specific to individual participants included in this study and not generalizable to broader Canadian culture. In order to assess other variables that might contribute to cultural fit, PCA loading factors, which are the weights used to calculate component scores, were extracted from SPSS and correlated with other demographic variables (Dressler et al., 2014).

## Results

### Cultural Consensus Analysis: What Social Values Contribute to Cultural Consensus and Fit?

Healthy Canadians were evaluated first to determine whether linguistic groups represented two distinct cultures within Canada or a distinct, unified culture. Migratory generations of Anglo-Canadians were evaluated second, to determine whether their heterogeneous background significantly contributed to cultural consensus. Third, patients with schizophrenia were evaluated to assess whether severe psychiatric disorders contribute to lower consensus. Finally, the combined contributions of familial migration and schizophrenia were assessed.

#### Linguistic Contributions to the SVS Cultural Domain in Healthy Canadians

All 147 Healthy Canadians with Canadian Parents were evaluated but the results did not achieve the 3:1 ratio threshold between first and second factors (2.81:1). When divided by linguistic group, Healthy Franco-Canadians with Canadian parents (3.12:1) and Healthy Anglo-Canadians with Canadian parents (3.04:1) displayed distinct models for the cultural social values domain. These results demonstrate that the social values of healthy Canadians are not homogenous and suggest that Anglo- and Franco-Canadians might have different social values.

#### Lower Consensus in More Recent Migratory Generations

Since the Healthy Anglo-Canadians with Canadian Parents cohort represented heterogeneous ancestral backgrounds, the contributions of migratory generation to cultural consensus were also assessed. The cohort of Healthy Canadians with Foreign Parents were combined with the Healthy Anglo-Canadians with Canadian Parents cohort and compared first as a single group and secondly as three separate groups according to second, third, and fourth+ migratory generations. As a unified group, Healthy Canadians with Foreign and Canadian Parents did not achieve cultural consensus (2.63:1). When evaluated separately by migratory generation, second (1.97:1) and third (2.82:1) generation Canadians did not achieve cultural consensus while fourth+ generation (3.03:1) Canadians did. These findings suggest that migratory generation contributes to cultural fit in the domain of social values, with later generations of Healthy Canadians with Foreign Parents sharing more social values with Healthy Anglo-Canadians with Canadian Parents.

#### Comparable Consensus in Patients With Schizophrenia

The contributions of mental health status were assessed by creating a cohort of Matched Patients-Controls with Canadians Parents. Since native language emerged as a defining feature of the cultural model for Healthy Canadians, the matched cohorts were first divided by linguistic group. Both groups achieved cultural consensus: Francophone Matched Patients-Controls with Canadian Parents (3.05:1) and Anglophone Matched Patients-Controls with Canadian Parents (3.17:1). Since consensus was achieved, further evaluation was not necessary. According to these results, patients share similar social values with healthy members of their linguistic group.

#### Combined Second-Generation and Patient Status Precludes Cultural Consensus

Matched Patients-Controls with Foreign Parents were also evaluated for cultural consensus by assessing first whether patients-only achieved consensus as a combined group with the patients from the above cohort of Matched Patients-Controls with Canadian Parents. Second, it was assessed whether the combined Matched Patients-Controls with Foreign Parents achieved consensus without the inclusion of participants with Canadian parents, and third it was assessed whether patients-only from the Matched Patients-Controls with Foreign Parents cohort achieved consensus without the inclusion of controls. Based on the previous findings, it was expected that this cohort would not achieve cultural consensus as a single group or when combined with matched controls, and the findings reflected this assumption. No grouping achieved consensus: combined with Anglo-Canadian Matched Patients + Controls (2.40:1), Matched Patients+ with Foreign Parents (2.20:1), as a single group Anglo-Matched Patients-Controls with Foreign Parents (1.04:1). These findings demonstrate that Patients with Foreign Parents heterogeneous social values when co-considered with Patients with Canadian Parents, Matched Controls with Foreign Parents, and other Patients with Foreign Parents.

#### Other Possible Determinants of Cultural Fit

Loading factor correlations were performed in Healthy Canadians with Canadian Parents to identify possible individual variations in cultural fit, such as secondary patterns attributable to demography or subcultural memberships, such as age, gender, and self-construal. Age and gender were not determinants of consensus. Residual agreement was driven by self-construal in both cohorts. SCS scores correlated with Factor 2 coefficients in Healthy Franco-Canadians with Canadian Parents (*r* = 0.283, *n* = 69, *p* = 0.018) and with Factor 1 coefficients in the Healthy Anglo-Canadians with Canadian Parents cohort (*r* = 0.348, *n* = 77, *p* = 0.002). These findings demonstrate that the correlation between SVS and self-construal differentially influences cultural fit in Healthy Franco- and Anglo-Canadians, suggesting that social orientation may play a different role than individual values. These findings agree with other studies by Crafa et al. (2018), which report that SVS and self-construal are distinct cultural domains despite being correlated (e.g., social interactions with peers alter the SVS and self-construals of individuals, but in different ways).

### Statistical Comparisons of Social Values: When do Franco- and Anglo-Canadians Diverge?

Independent samples t-tests revealed that Healthy Franco-Canadians with Canadian Parents valued being *harmonious* and *indirect* but also *assertive* and *vocal*. Healthy Anglo-Canadians with Canadian Parents distinctly reported not valuing *assertiveness*, *vocalness*, *indirectness* but marginally valued *individualistic* and *solitary*. Figure 1, Table 2 depicts the complete statistical results and average ratings of social values adjusted against the “answer key” for shared background for Healthy Franco- and Anglo-Canadians with Canadian Parents.

## Discussion

### Summary of Main Findings

Healthy Franco- and Anglo-Canadians represent two linguistic groups that have different social values despite residing within the same country. Within the Anglo-Canadian cohort, migratory generation contributes substantially to cultural consensus and fit. When second generation migrants are included in the CCA, consensus is less homogeneous. Closer examination reveals greater heterogeneity of values in second and third generations of Canadians, while fourth and later migratory generations achieve consensus with a standard sample size. According to the most recent national census, a majority of Canadians (56%) speak English as their primary language compared to French (21%), and Anglo-Canadian culture is dominant in most provinces outside Quebec (Statistics Canada, 2016). These findings suggest that the dominant culture in Canada may be relatively heterogeneous in their social values even among natural born Canadians.

Canadian culture diverged around key social values. Healthy Franco-Canadians reported valuing both social harmony and self-assertion while Healthy Anglo-Canadians valued individualism and solitude. In this respect, Healthy Franco-Canadians appear to be defined more prominently by modalities of communication while Healthy Anglo-Canadians are more concerned with self-definition and differentiation.

Interestingly, age and gender did not contribute to cultural fit in the domain of social values, but self-construal did. Healthy Franco-Canadians showed a subcultural division (factor 2) by independence and interdependence while Healthy Anglo-Canadians showed a primary division (factor 1) according to these social orientations. These findings are logical considering the correlation between the SVS and self-construal, as well as the fact that Anglo-Canadians varied by migratory generation (i.e., showed signs of cultural divisions) while Franco-Canadians were not evaluated in these terms (i.e., any divisions should be subcultural).

Contrary to outcomes of previous research, patients with schizophrenia were good fits for their cultural group as long as they were third generation Canadians or later. These findings are particularly noteworthy because patients were substantially over-represented in this study (50% of CCA sample compared to 1% in the Canadian population; Stewart, 2002; Dealberto, 2010). Impaired social skills that are characteristic of schizophrenia do not appear to preclude cultural learning in average patients.

Second generation Canadians with schizophrenia showed lower consensus compared to all other cohorts studied, with a ratio of nearly 1:1. Numerous research studies have reported greater prevalence and severity of psychiatric disorders in second generation citizens, and some studies suggest that these rates are caused by poor social adaptation or to unique social challenges of navigating a bicultural upbringing (Bourque et al., 2011). Our findings empirically support these suppositions by demonstrating that patients in this generation are relatively heterogeneous in the social values domain of culture, which may contribute to feelings of alienation or marginalization (Berry and Sabatier, 2010).

### Implications for Intersectional, Intracultural, and Patient Studies

Across much cross-cultural literature, culture is often conflated with country, ancestry (and its synonyms, like race or ethnicity), and linguistic group (Cohen, 2009). The proper approach to defining culture is subject of much debate (Hofstede, 1980; Hudelson, 2004). The findings from this study help to parse differences between such terms. Taken together, findings demonstrate that linguistic groups constitute distinct cohorts in the domain of social values. They also show that ancestry contributes to cultural consensus but is not the sole determinant.

By pointing to a complex interaction between culture and language that agrees with an emerging body of literature on the role of language in cultural construction and meaning-making, these findings challenge notions that familial ancestry is the primary contributor to cultural affiliation and that language is a secondary contributor (Kramsch and Widdowson, 1998; Holliday, 2010; Liwei, 2010; Horowitz, 2012). They also demonstrate that culture is, in part, co-constructed from country, ancestry, and language, and that each of these characteristics ought to be considered when defining a cultural group for a quantitative study.

From this perspective, we propose defining cultural membership as a co-construction between multiple traits that ought to be assessed as a standard procedure when evaluating quantitative data. This definition of culture embraces an intersectional approach to sample characterization by quantifying consensus rather than assuming cultural homogeny. Many countries house natural born citizens with diverse ancestral and linguistic backgrounds and empirical research often yields heterogeneous or contradictory findings (Freese and Peterson, 2017; Shrout and Rodgers, 2017). Socially diverse study cohorts may partially explain these disagreements. We propose that studies examining cultural modalities, such as social values, should evaluate whether their study sample achieves cultural consensus as a routine part of data analyses. In fact, since many countries house multiple cultural groups and multiple ancestral backgrounds, this characterization is highly beneficial for validating a study sample. This suggestion may be especially important for patient studies, since the lowest consensus emerged in this cohort. Clinical researchers face unique challenges when recruiting patients, who can be hard to find and retain in a study. Accordingly, clinical research studies often include smaller sample sizes, which may mean less sample agreement and also greater chances for nonreplicable findings (Pashler and Wagenmakers, 2012; Pashler and Harris, 2012). Considering key sociodemographic variables, such as language and ancestry, may improve reproducibility through optimizing sample construction.

### Applications of the Top-Down Approach

The term *culture* is ill-defined in many research studies, and the top-down CCA used by this and other studies offers a practical approach for defining culture based on a random sample. Whereas most CCA methods use a bottom-up approach that involves, first, handpicking study participants according to predefined cultural demographics and intersubject agreement and, second, extracting a cultural model from the final sample. The top-down approach applied by the present study used random participant samples and sample matching to evaluate whether a group represented the culture being ascribed to them. This approach can be adapted to characterize study samples and verify that cohorts truly reflect their assigned cultural groups by more precisely describing agreement. The culture of a study population may be pragmatically defined as consensus between a sample for which inclusion criteria for native language, birth country and familial ancestry has been recorded and deliberately delineated.

### Precautions that Avoid Common CCA Pitfalls

The top-down application of CCA used in the present study arguably circumvents common criticisms of the CCA approach. In particular, CCA is sometimes criticized for defining culture according to an abstract ideal built according to group consensus, which additionally assumes that group members are experts in their own culture (Aunger, 1999). This criticism, of course, raises the question of what is being measured and to what degree of specificity. Defining *culture* according to an abstract ideal is problematic for many reasons, not least of all because cultural idealism risks implying superiority over other cultural groups (Wagner, 2016). Inherent in the CCA design is the attempt to circumvent cultural idealism. CCA uses the participant group to dictate the items of consensus rather than imposing cultural traits on the analyses. Assuming group members are cultural experts is problematic because their cultural fit can vary widely.

However, cultural fit is assessed by CCA as well, and individual variations can be evaluated. Additionally, we argue that using CCA to characterize sample groups is intrinsically better than assuming that a sample from the same country, ancestry, or linguistic group is inherently representative of culture, as many studies currently do. Nonetheless, heeding these criticisms has no inherent risks, such as Type I or II statistical errors, due to the methodology, but does provides a more thorough interrogation of the data.

In order to ensure that the presented CCA results are validly interrogating the research questions, the present study took several precautions. First, SVS was used instead of self-construal, in order to evaluate more dimensions of social values as a cultural domain and theoretically yield a more nuanced depiction of culture. Second, participants were evaluated according to more than one cultural dimension, i.e., their linguistic, ancestral, and self-construal groups were evaluated for logical and precedented agreements, resulting in a thorough characterization of the study population. Third, matched cohorts were included whenever a sample size was smaller than 30 to act as a standard for minimum group size.

### Limitations and Future Directions

This study had several limitations. Firstly, Likert rating scales can magnify individual differences if participants interpret the scores differently, and different measures and domains may yield agreement differences. CCA outcomes examining values schemas additionally can be assessed using free-response or multiple choice instead of Likert ratings. Culture can be defined across multiple domains, and SVS is just one of them. Future studies should examine other cultural domains, such as religious and political identities, to assess variations in cultural consensus. Secondly, although Franco-Canadians may descend from French colonists or migrants from French territories, such as Haiti or Martinique, Franco-Canadians with migrant parents did not respond to study advertisements. This was unsurprising since most French Canadians’ families emigrated much earlier than Anglo-Canadians, However, having foreign parents was not exclusionary for this cohort and the contributions of migratory generation to the broader Franco-Canadian population could not be assessed. Thirdly, like much clinical research, this study evaluated a limited number of patients due to practical constraints of time, resources, and availability. As a result, all patient cohorts included samples smaller than 30, which are suboptimal. This limitation was offset by the matched control group, which also achieved cultural consensus. Additionally, third and later generation patients achieved cultural consensus with their matched control cohorts despite being over-represented in this CCA. The low agreement in second-generation patients was nearly 1:1, suggesting that agreement might not be achieved even with a very large sample size. Finally, all patients were high functioning enough to participate in a research study. Thus, they represent the average studied patient population, but they do not reflect extremely severe patient with schizophrenia. Moreover, we only included a schizophrenia sample, and findings may be generalizable to some but not all psychiatric disorders. Future studies should evaluate whether other psychiatric populations, such as those diagnosed with autism, exhibit cultural consensus.

## Conclusions

In conclusion, this study uses top-down CCA to evaluate whether language, ancestry, and mental health status contribute to cultural membership or fit. Specifically, the current study showed that cultural consensus was achieved by language and migratory generation yet not by mental health in third and later migratory generations. These findings suggest that second generation migrants with schizophrenia display poor cultural fit, which may explain other studies reporting severe distress in these populations. The use of top-down CCA appears to be an effective way to characterize study cultural membership and fit samples, and provides a practical way to empirically define the degree of homogeneity of participants’ cultural affiliations.

## Author Contributions

DC conceived of the study, designed the materials and methods, recruited and evaluated the study populations, and wrote the paper. JL performed the statistical analyses, assisted with data collection and study population evaluations, and provided valuable insights that were incorporated into this paper. MB supervised and funded this project and provided feedback on the creation of study materials, statistical analyses, and final manuscript.

### Conflict of Interest Statement

The authors declare that the research was conducted in the absence of any commercial or financial relationships that could be construed as a potential conflict of interest.

## Footnotes

^1^https://www.projet-pfc.net/

^2^https://corpus.byu.edu/can/
